# Detection of Overlooked Rare *EGFR* Mutations in Non‐small Cell Lung Cancer Using Multigene Testing

**DOI:** 10.1111/1759-7714.70007

**Published:** 2025-02-13

**Authors:** Naoki Shiraishi, Takayuki Takahama, Kazuko Sakai, Kaoru Tanaka, Yuzuki Nakagawa, Hiroaki Kanemura, Tomohiro Nakayama, Yusuke Kawanaka, Takashi Kurosaki, Shinichiro Suzuki, Tsutomu Iwasa, Junko Tanizaki, Chiaki Inagaki, Kimio Yonesaka, Kazuya Fukuoka, Tetsuya Mitsudomi, Kazuto Nishio, Hidetoshi Hayashi, Kazuhiko Nakagawa

**Affiliations:** ^1^ Genome Medical Center Kindai University Hospital Osaka Japan; ^2^ Department of Medical Oncology Kindai University Faculty of Medicine Osaka Japan; ^3^ Department of Genome Biology Kindai University Faculty of Medicine Osaka Japan; ^4^ Department of Medical Oncology Kishiwada City Hospital Osaka Japan; ^5^ Clinical Research Center Kindai University Hospital Osaka Japan; ^6^ Izumi City General Hospital Osaka Japan; ^7^ Faculty of Medicine Kindai University Hospital Sayama Japan; ^8^ Cancer Center Kindai University Hospital Osaka Japan

**Keywords:** *EGFR* mutation, next‐generation sequencing, non‐small cell lung cancer, Oncomine Dx Target Test

## Abstract

**Background:**

Recognizing rare molecular variants of driver mutations poses a challenge in precision oncology, particularly for treatment of non‐small cell lung cancer (NSCLC). In this study, we aimed to determine whether Oncomine Dx Target Test Multi‐CDx System (ODxTT), the most widely used genetic test for NSCLC in Japan, potentially overlooks druggable *EGFR* mutations.

**Materials and Methods:**

Among 418 patients who underwent molecular testing using ODxTT at our hospital, 267 were diagnosed with adenocarcinoma. No mutations were reported in 82 of these cases. For these 82 cases, we searched for *EGFR* mutations in exons 18–21 by examining the binary alignment map file. Once a mutation was identified, its pathological significance was evaluated using the ClinVar database to determine whether ODxTT had overlooked any actionable *EGFR* mutations.

**Results:**

Mutations in *EGFR* exons 19 and 18 were identified in six and four cases, respectively. Three, six, and none of these variants were detectable using the Cobas EGFR Mutation Test v2, Lung Cancer Compact Panel, and Amoy Dx, respectively. Of the 10 patients, five were subsequently treated with EGFR TKI; three showed partial response, one had stable disease, and one had progressive disease.

**Conclusions:**

ODxTT failed to identify 10 actionable *EGFR* mutations, accounting for 12.2% (10/82) of the cases initially reported as not carrying actionable mutations. Therefore, comprehensive genomic profiling should be actively performed early in cases with high clinical suspicion of *EGFR* mutations.

## Graphical abstract

We reviewed BAM data of 82 patients who underwent ODxTT and were initially reported as negative. ODxTT failed to identify 10 actionable EGFR mutations, accounting for 12.2% (10/82) of the cases. Three of these variants were detectable using Cobas EGFR Mutation Test v2, six variants were detectable using Lung Cancer Compact Panel, and none were detectable using Amoy Dx. Of the 10 patients, five were subsequently treated with EGFR TKI; three showed partial response, one had stable disease, and one had progressive disease. Comprehensive genomic profiling should be actively performed early in cases with high clinical suspicion of EGFR mutations.

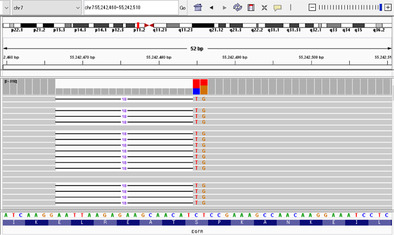

## 1 Introduction

The terms “personalized medicine” and “tailored treatment” have been integral to cancer therapy for many years. Since the 2000s, new therapeutic approaches, such as molecular‐targeted therapies and immune checkpoint inhibitor administration, have emerged, demonstrating greater efficacy than traditional cytotoxic anticancer agents [1, 2, 3]. Companion diagnostics are co‐developed with therapeutic drugs to aid in selecting patients who are most likely to benefit based on their biological characteristics that predict response.

In non‐small cell lung cancer (NSCLC), several genetic mutations influencing therapy choices have been identified, including mutations in *EGFR*, *ALK*, *ROS1*, and *BRAF* [4, 5, 6, 7, 8, 9, 10, 11]. Many molecularly targeted therapies inhibit aberrant protein production resulting from driver mutations or translocations that directly contribute to cancer development. Traditionally, these mutations and fusion genes have been tested using single assays [12, 13, 14, 15].

The advent of next‐generation sequencing (NGS) has enabled simultaneously identifying multiple driver cancer genes. Oncomine Dx Target Test (ODxTT) is an NGS panel for NSCLC testing approved in Japan in February 2019 and serves as a companion diagnostic tool that identifies genetic alterations using DNA and RNA isolated from formalin‐fixed, paraffin‐embedded (FFPE) tumor samples. This test can detect 46 cancer driver gene variants, including *EGFR* mutations (such as L858R, T790M, and exon 19 deletions); *BRAF*, *KRAS*, *ERBB2*, and *MET* exon 14 skipping mutations; and *ALK*, *ROS1*, *RET*, and *NTRK1/2/3* fusions. ODxTT offers several advantages, including fast turnaround time, reduced labor for specimen processing, and low tissue consumption.

Despite improved success rates nationwide, significant differences in mutation detection rates persist between institutions [16]. *EGFR* mutants are common driver oncogenes in NSCLC in East Asia, accounting for approximately 50% of lung adenocarcinomas [17]. With the development of numerous molecularly targeted drugs, accurately detecting *EGFR* mutations is crucial for effective treatment of lung cancer. Failure to obtain precise test results may lead to missed opportunities for implementing appropriate molecular‐targeted therapies for advanced NSCLC. Various studies have examined the success rate of ODxTT and differences in mutation detection rates [16], as well as the positivity rate of Cobas, a PCR‐based *EGFR* singleplex test [18, 19]. *EGFR* mutations have also been missed by Cobas but detected using comprehensive genomic profiling (CGP) [20].

No study has specifically reported on the type and frequency of potentially missed driver genes in the ODxTT analysis pipeline in clinical practice. Therefore, in this study, we aimed to summarize and report *EGFR* mutations that were not identified as positive by ODxTT but were detectable by reviewing binary alignment map (BAM) data. The results are anticipated to provide insight into the development of a new analytical pipeline.

## 2 Materials and Methods

### 2.1 Gene Mutation Testing

Specimens used for companion diagnostics were properly processed during the pre‐analytical stage. Tissue handling and fixation of excised specimens were performed using 10% neutral buffered formalin for fixation times of 6–48 h, following “Lung Cancer Treatment Regulations, 8th Edition” and “Regulations for Handling Histopathology Specimens for Genomic Medicine” [21, 22, 23]. ODxTT is an in vitro diagnostic NGS method used for detecting somatic alterations in human DNA and RNA isolated from FFPE NSCLC tissues. Macrodissection was performed to obtain a tumor percentage of at least 30%. The extracted DNA and RNA were found to be at concentrations of at least 0.83 and 1.43 ng/μL, respectively. Following sequencing, DNA libraries should have exhibited a coverage of at least 347 for each variant to be considered undeterminable. Similarly, RNA libraries should have exhibited a total of at least 5000 mapped reads to be considered undeterminable. ODxTT was performed using an Ion Torrent PGM Dx NGS system.

The ODxTT system workflow was conducted using Ion Torrent Dx FFPE Sample Preparation Kit, Ion PGM Dx Library Kit, Ion One Touch Dx Template Kit, and Ion PGM Dx Sequencing Kit, following the manufacturer's instructions. The pathology department of the hospital received ISO 15189 certification in December 2016. The Genome Medical Center received ISO 15189 certification in August 2023, and the specimens were managed according to these standards.

### 2.2 Clinical Information

Clinical data were collected for several parameters. The specimen information included sex; patient age at specimen collection; histology; results for *EGFR*, *ALK*, *ROS1*, *RET* fusion, *ERBB2*, and *BRAF* (p.V600E) mutations; date of specimen collection; tumor percentage assessment; macrodissection performed; specimen collection method; and extracted DNA and RNA concentrations.

### 2.3 Method

Patients with a pathologically confirmed diagnosis of NSCLC and for whom a BAM file was available from June 2020 to August 2023 with 46 genes tested at our institution using ODxTT were eligible for inclusion in the study (Figure S1). Written informed consent was obtained from all patients. This study was approved by the Institutional Review Board of Kindai University (approval date: October 25, 2023; IRB number: R05‐120). The trial was registered in the UMIN database with ID 000054164.

The BAM file is a binary format file in which the reads sequenced by NGS are aligned and mapped to a reference sequence. The sequence reads were aligned to the hg19 human reference genome based on GRCh37.p5 assembly, which is preinstalled in the Ion Torrent PGM Dx NGS system. The Integrated Genomics Viewer (IGV, Broad Institute, Regents of the University of California) was used for visual inspection [24]. BAM data and index files from patients pathologically diagnosed with adenocarcinoma, in which no mutations were detected in either DNA or RNA, were downloaded from Torrent Suite DX and visually inspected with regions chr7:55241640 to 55 241 720 in the Ex18 region, chr7:55242400 to 55 242 500 in the Ex19 region, and chr7:55259510 to chr7:55259520 in the Ex21 region. Ex20 insertions were not included in the observation target. Genetic variants, such as nucleotide substitutions and insertions/deletions, were identified using COSMIC. The pathological significance was assessed using ClinVar. The best overall response for patients with driver gene mutations identified in this way and treated with EGFR TKIs was evaluated. Definitions of the best therapeutic response and disease control were based on RECIST v1.1 criteria, with response defined as complete response (CR) or partial response (PR), and disease control defined as CR, PR, or stable disease (SD).

## 3 Results

From June 2020 to August 2023, ODxTT was performed on 418 patients after in‐house production. The baseline characteristics of the patients are summarized in Table 1. The median age was 73 (range 29–93), with 282 patients (67.4%) being male. Of the total, 267 patients had adenocarcinoma. The success rate of the Oncomine Dx Target Test was 98.8% (413/418). Five cases did not meet the QC index after DNA or RNA sequencing, rendering them undeterminable. Macrodissection was conducted in 86.1% (360/418) of cases, with tumor cell percentages exceeding 30%. The most common procedure performed was endobronchial ultrasonography with a guided sheath (EBUS‐GS), accounting for 45.7% of the cases (191/418); other procedures included endobronchial ultrasound‐guided transbronchial needle aspiration (EBUS‐TBNA) (16.7%, 70/418); CT‐guided biopsy (3.8%, 16/418); and surgical resection (27.9%, 117/418).

**TABLE 1:** Patient characteristics.

		Total *n* = 418
Median age (range)		73 (29–93)
Sex, *n* (%)	Male	282 (67)
	Female	136 (33)
Histology, *n* (%)	Adenocarcinoma	205 (49)
	NSCLC favor Adenocarcinoma	62 (15)
	Squamous cell carcinoma	61 (15)
	NSCLC favor Squamous cell carcinoma	37 (9)
	NOS	42 (10)
	Other	11 (3)
Sample, *n* (%)	TBB	191 (46)
	EBUS‐TBNA	70 (17)
	CTGNB	16 (4)
	Pleura biopsy	16 (4)
	Cell block	8 (2)
	Surgical resection, primary	84 (20)
	Surgical resection, metastasis	33 (8)

Abbreviations: CTGNB, computed tomography‐guided needle biopsies; EBUS‐TBNA, endobronchial ultrasound‐guided transbronchial needle aspiration; NSCLC, non‐small‐cell carcinoma; NOS, not otherwise specified; TBB, transbronchial biopsy.

**FIGURE 1:**
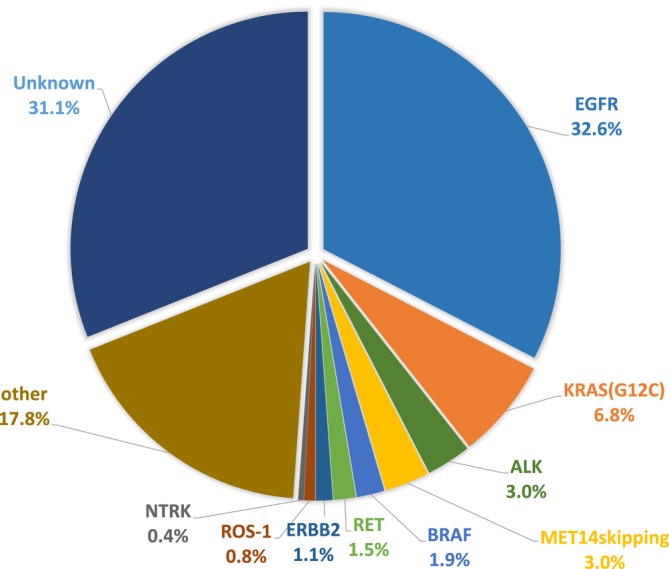
Driver mutations in *EGFR* detected using ODxTT in adenocarcinoma (*n* = 264).

The detection rates for mutations in the 264 patients with adenocarcinoma, excluding the three cases in which the mutations could not be determined by DNA or RNA, are shown in Figure 1, with details of the *EGFR* mutations presented in Table 2. Mutations in *EGFR*, *KRAS* (p.G12C), *BRAF* (p.V600E), *ALK* fusion, and *MET14* skipping were detected in 86 (32.6%), 18 (6.8%), 5 (1.9%), 8 (3.0%), and 8 (3.0%) cases, respectively. Additionally, *RET* fusion was detected in 4 (1.5%) of cases, whereas *ERBB2*, *ROS1* fusion, and *NTRK* fusion were detected in 3 (1.1%), 2 (0.8%), and 1 (0.4%) of cases, respectively, with other mutations found in 47 (17.8%) cases. No mutation was detected in 82 cases (31.1%). The BAM data of these 82 cases were downloaded and visually checked using IGV in the ranges of chr7:55241640–55241720, chr7:55242400–55242500, and chr7:55259510–55259520.

**TABLE 2:** Details of the EGFR mutations detected by the ODxTT.

Genotypes	Subtypes			*n*
EGFR	L858R			40
	Exon 19 deletion	c.2235_2249delGGAATTAAGAGAAGC	COSM6223	14
		c.2236_2250delGAATTAAGAGAAGCA	COSM6225	10
		c.2237_2251delAATTAAGAGAAGCAA	COSM12678	4
		c.2240_2254delTAAGAGAAGCAACAT	COSM12369	3
		c.2240_2257delTAAGAGAAGCAACATCTC	COSM12370	2
		c.2237_2255delAATTAAGAGAAGCAACATCinsT	COSM12384	2
		c.2239_2251delTTAAGAGAAGCAAinsC	COSM12383	1
	Exon 20 Insertion			3
	L861Q			1
	L861Q + G719S			1
	G719A + E709A			1
	G719A + E709K			1
	G719A + S768I			1
	T790M			1

Abbreviation: EGFR, epithelial growth factor receptor.

**FIGURE 2:**
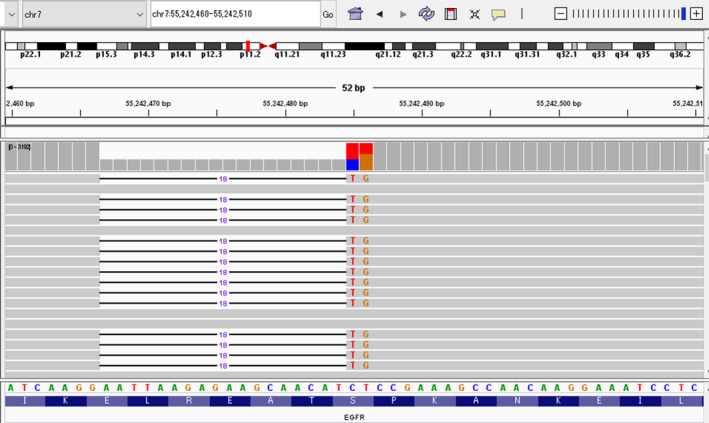
BAM data for *EGFR* exon 19 deletion (p.E746_S752delinsV) displayed in IGV. The chr7:55242400–55 242 500 region in case 9 was visualized using IGV. Although the patient tested negative, a deletion was observed.

Ten druggable *EGFR* mutations were found in these 82 cases: four E709_T710 delinsD in exon 18 and six deletions in exon 19. No undetected mutation was found in the exon 21 region [25]. BAM data showing the *EGFR* exon 19 deletion (p.E746_S752delinsV) with IGV are presented in Figure 2. Details of the detected *EGFR* mutations are shown in Table 3. Among the seven variants, three were detectable using Cobas and six using Lung Cancer Compact Panel, and all were undetectable using Amoy Dx. Six variants included deletions and insertions.

**TABLE 3:** Details of the overlooked EGFR mutations.

	AA mutation	CDS mutation	VAF(%)	SEX	Age	Smoking	ClinVar	COSMIC ID	Other assay	TKI response
Case 1	p.E709_T710delinsD	c.2127_2129del	25.1	Male	71	Ex‐smoker	Pathogenic	COSM51525	Cobas:× LCCP:× Amoy:×	—
Case 2	p.E709_T710delinsD	c.2127_2129del	32.3	Male	65	Current smoker	Pathogenic	COSM51525	Cobas:× LCCP:× Amoy:×	—
Case 3	p.E709_T710delinsD	c.2127_2129del	48.9	Male	50	Ex‐smoker	Pathogenic	COSM51525	Cobas:× LCCP:× Amoy:×	Afatinib PD
Case 4	p.E709_T710delinsD	c.2127_2129del	38.1	Female	48	Never	Pathogenic	COSM51525	Cobas:× LCCP:× Amoy:×	Investigational EGFR‐TKI SD
Case 5	p.E746_T751delinsV	c.2237_2252delinsT	26.6	Female	43	Current smoker	No registration	COSM12386	Cobas:〇 LCCP:〇 Amoy:×	Osimertinib PR
Case 6	p.T751_I759delinsN	c.2252_2276delinsA	51.1	Female	56	Never	No registration	COSM96856	Cobas:× LCCP:〇 Amoy:×	—
Case 7	p.S752_I759del	c.2253_2276del	6.2	Female	72	Never	Drug response	COSM13556	Cobas:〇 LCCP:〇 Amoy:×	—
Case 8	p.E746_T751delinsVP	c.2237_2251delinsTTC	22.8	Male	65	Ex‐smoker	Drug response	COSM18421	Cobas:× LCCP:〇 Amoy:×	Osimertinib PR
Case 9	p.E746_S752delinsV	c.2237_2256delinsTG	60.7	Female	80	Ex‐smoker	No registration	COSM674057	Cobas:× LCCP:〇 Amoy:×	—
Case 10	p.E746_P753delinsVS	c.2237_2257delinsTCT	28.8	Male	73	Ex‐smoker	Drug response	COSM18427	Cobas:〇 LCCP:〇 Amoy:×	Osimertinib PR

*Note:* EGFR‐TKI not administered.

**FIGURE 3:**
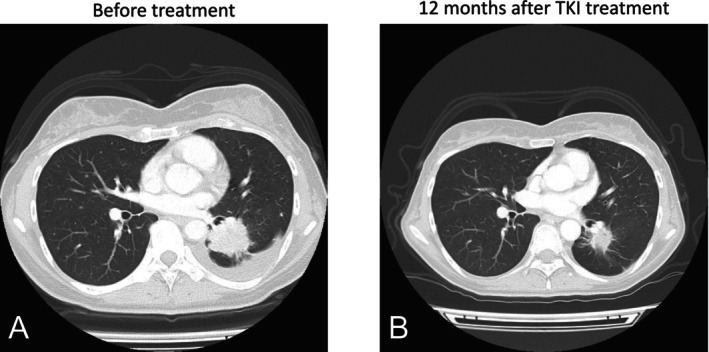
Chest computed tomography images before and after administering osimertinib in case 5. (A) Chest CT scan before osimertinib treatment. (B) Chest CT scan 1 year after treatment. Tumor shrinkage and decreased pleural effusion were observed.

Of these 10 patients, five received EGFR TKI treatment based on the study results; three patients had PR, and one patient had SD as the best therapeutic response. For example, a 43‐year‐old Japanese woman diagnosed with stage IV NSCLC in May 2021 was initially negative for *EGFR* mutations by ODxTT. She started a combination therapy of carboplatin, pemetrexed, and pembrolizumab on June 21, 2021, yielding SD as the best response. Subsequent analysis of the ODxTT BAM file revealed a PCR‐detectable *EGFR* mutation, confirmed by Cobas real‐time PCR as an *EGFR* exon 19 deletion. Treatment with osimertinib (80 mg) was initiated on February 1, 2023, 3 months after the last dose of pembrolizumab to avoid the risk of drug‐induced pneumonitis. A CT scan on day 77 of treatment showed significant reduction in the primary tumor and metastases (Figure 3). After a year of treatment, the patient achieved PR. As of February 2024, treatment with osimertinib continued without significant adverse events or tumor progression.

## 4 Discussion

Our study is the first to summarize the detection of *EGFR* mutations potentially missed by the ODxTT over a three‐year period by using a review of in‐house BAM data. We identified four *EGFR* exon 18 mutations and six *EGFR* exon 19 deletions in 82 adenocarcinoma patients initially reported as having no genetic mutations. This implies a 12.2% miss rate (10 out of 82 cases) for *EGFR* mutations. These cases were reported as negative rather than no‐calls. Many facilities retest with a single‐gene test if a no‐call result occurs but may not retest if the result is negative, leading to missed treatment opportunities.

For all variants detected in this study, the variant allele frequency was sufficient compared with the limit of detection, indicating that these false negatives were not due to low pathological tumor percentages but rather because they were not included in the call list of the analysis program. Such false negatives could occur in many facilities performing macrodissection. Therefore, determining the variants that each test method can detect is crucial before selecting a test method. Minor *EGFR* mutations detected here will hopefully be included in the call list, and ODxTT is anticipated to detect additional EGFR mutations in the future.

NGS testing is essential for determining cancer treatment strategies [26]. Considering that NGS might yield false‐negative results in cases with low tumor fractions, many facilities are improving macrodissection and accurate tumor fraction calculation [27, 28]. However, many institutions report lower *EGFR* mutation detection rates for ODxTT than for existing singleplex tests [16]. At our institution, the *EGFR* mutation detection rate for adenocarcinomas was 32%, which was lower than previously reported data [17, 29]. The current study suggests that rare mutations contribute to these low detection rates.

Several clinical trials now provide data on rare driver gene mutations, and a standard of care using molecular‐targeted therapies is being established [30, 31]. Therefore, appropriate measures must be taken to detect variants potentially missed by conventional assays.

This study has several limitations. Firstly, not all variants detected in the ODxTT BAM data were confirmed by any other genetic test. However, these variants are listed as detectable in the package inserts of other genetic tests. Further validation of these results using additional methods, such as functional studies or prospective clinical trials, would enhance confidence of the data. Secondly, we did not investigate the response of these undetected variants to EGFR TKIs in all patients. Additionally, this was a relatively small single‐center retrospective study. Patient selection bias should also be considered in this study because only patients with negative ODxTT results were studied. Additionally, reanalysis of the BAM files by visual inspection may have detection bias, which limits the applicability of the results obtained here to a broad population and different settings.

Considering these limitations, future studies should examine additional patients with NSCLC at multiple centers to further validate findings across different centers and patient populations and reach robust conclusions.

No multigene or single‐gene test could detect all the mutations found in this study (Table 4). Amplicon‐based NGS and PCR assays can only detect variants using pre‐designed primers. CGP uses a hybrid capture method, which theoretically can detect all mutations. *EGFR* mutations missed by singleplex tests but detected by CGP have been reported [20]. Initial genetic testing oversights have been reported for EGFR mutations, as well as for other oncogenes associated with NSCLC treatment [32, 33]. CGP testing may detect driver gene mutations other than EGFR that were not detected by first‐line gene test, thereby expanding therapeutic strategies. Additionally, it may detect genetic mutations for which there are approved drugs for other types of cancer, and may expand therapeutic strategies such as clinical trials. Therefore, CGP (which is covered by insurance in Japan) should be aggressively performed on patients with negative first‐line gene test results.

**TABLE 4:** List of EGFR mutations detectable by various CDx tests.

Exon No.	Hotspot ID	Amino acid change	Nucleotide change	Cobas	ODxTT	Amoy	LCCP	CGP
18	COSM12988	p.E709K	c.2125G>A		C		C	〇
18	COSM13427	p.E709A	c.2126A>C		C		C	〇
18	COSM13009	p.E709G	c.2126A>G		C		C	〇
18	COSM12371	p.E709V	c.2126A>T		C		C	〇
18	COSM116882	p.E709Q	c.2125G>C				C	〇
18	COSM12428	p.E709H	c.2125_2127delinsCAT				ref	〇
18	COSM51525	p.E709_T710delinsD	c.2127_2129del					〇
18	COSM1169617	p.L718V	c.2152C>G		ref		ref	〇
18	COSM6503269	p.L718Q	c.2153T>A		ref		ref	〇
18	COSM6252	p.G719S	c.2155G>A	C	C	C	C	〇
18	COSM6253	p.G719C	c.2155G>T	C	C	C	C	〇
18	COSM18425	p.G719D	c.2156G>A		C		C	〇
18	COSM6239	p.G719A	c.2156G>C	C	C	C	C	〇
19	COSM87245	p.I744T	c.2231TdelinsC		ref		ref	〇
19	COSM26038	p.K745_E749del	c.2233_2247delAAGGAATTAAGAGAA	C	C		C	〇
19	COSM1190791	p.K745_A750delinsT	c.2234_2248delAGGAATTAAGAGAAG		C		C	〇
19	COSM28517	p.E746_E749del	c.2235_2246delGGAATTAAGAGA		C		C	〇
19	COSM13550	p.E746_A750delinsIP	c.2235_2248delinsAATTC	C			C	〇
19	COSM6223	p.E746_A750del	c.2235_2249delGGAATTAAGAGAAGC	C	C	C	C	〇
19	COSM13552	p.E746_T751delinsIP	c.2235_2251delinsAATTC	C			C	〇
19	COSM13551	p.E746_T751delinsI	c.2235_2252delinsAAT	C	C	C	C	〇
19	COSM12385	p.E746_S752delinsI	c.2235_2255delinsAAT	C			C	〇
19	COSM6225	p.E746_A750del	c.2236_2250delGAATTAAGAGAAGCA	C	C	C	C	〇
19	COSM12728	p.E746_T751del	c.2236_2253delGAATTAAGAGAAGCAACA	C	C	C	C	〇
19	COSM12678	p.E746_T751delinsA	c.2237_2251delAATTAAGAGAAGCAA	C	C	C	C	〇
19	COSM12386	p.E746_T751delinsV	c.2237_2252delinsT	C			C	〇
19	COSM12416	p.E746_T751delinsVA	c.2237_2253delinsTTGCT	C	C		C	〇
19	COSM12367	p.E746_S752delinsA	c.2237_2254del18	C		C	C	〇
19	COSM12384	p.E746_S752delinsV	c.2237_2255delinsT	C	C	C	C	〇
19	COSM18427	p.E746_P753delinsVS	c.2237_2257delinsTCT	C			C	〇
19	COSM12422	p.L747_A750delinsP	c.2238_2248delinsGC	C	C	C	C	〇
19	COSM12419	p.L747_T751delinsQ	c.2238_2252delinsGCA	C	C	C	C	〇
19	COSM23571 (COSM12369)	p.L747_T751del	c.2239_2253del	C	C	C	C	〇
19	COSM12403	p.L747_S752delinsQ	c.2239_2256delinsCAA	C		C	C	〇
19	COSM6220	p.E746_S752delinsD	c.2238_2255delATTAAGAGAAGCAACATC	C	C	C	C	〇
19	COSM6218	p.E746_R748del	c.2239_2247delTTAAGAGAA	C	C	C	C	〇
19	COSM12382	p.L747_A750delinsP	c.2239_2248delinsC	C	C	C	C	〇
19	COSM12383	p.L747_T751delinsP	c.2239_2251delinsC	C	C	C	C	〇
19	COSM6254 (=COSM12369)	p.L747_T751del	c.2239_2253del15	C	C	C	C	〇
19	COSM6255	p.L747_S752del	c.2239_2256delTTAAGAGAAGCAACATCT	C	C	C	C	〇
19	COSM12387	p.L747_P753delinsQ	c.2239_2258delinsCA	C	C	C	C	〇
19	COSM6210	p.L747_T751delinsS	c.2240_2251delTAAGAGAAGCAA	C	C	C	C	〇
19	COSM12369	p.L747_T751del	c.2240_2254del	C	C	C	C	〇
19	COSM12370	p.L747_P753delinsS	c.2240_2257delTAAGAGAAGCAACATCTC	C	C	C	C	〇
19	COSM13556	p.S752_I759del	c.2253_2276del	C			C	〇
19	COSM26441	p.L747_S752delinsQ	c.2238_2256delinsGCAA			C	C	〇
19	COSM133186	p.I744_E749delinsLKR	c.2230_2246delinsCTTAAGAG				C	〇
19	COSM85798	p.I744_A750delinsVK	c.2230_2249delinsGTCAA				C	〇
19	COSM221565	p.K745_A750del	c.2232_2249del				C	〇
19	COSM3734668	p.E746_A750del	c.2232_2249delinsAAA				C	〇
19	COSM12423	p.I740_K745dup	c.2214_2231dup				C	〇
19	COSM24869	p.E746_T751del	c.2235_2252del				C	〇
19	COSM18420	p.E746del	c.2235_2237del				C	〇
19	COSM13549	p.E746_T751delinsA	c.2235_2251delinsAG				C	〇
19	COSM6506513	p.E746_T751delinsFPS	c.2235_2251delinsATTCCCGT				C	〇
19	COSM255152	p.K745_E746insTPVAIK	c.2234_2235insAACTCCCGTCGCTATCAA				C	〇
19	COSM51504	p.I740_K745dup	c.2217_2234dup				C	〇
19	COSM133189	p.E746_S752del	c.2236_2256del				C	〇
19	Su2017	p.E746_A751delins*fs	c.2236_2249del				C	〇
19	COSM133187	p.E746_T751delinsL	c.2236_2252delinsCT				C	〇
19	COSM3727812	p.E746_L747delinsNY	c.2236_2241delinsAATTAT				C	〇
19	COSM12413	p.E746_A750delinsRP	c.2236_2248delinsAGAC				C	〇
19	COSM26680	p.E746_T751delinsI	c.2236_2252delinsAT				C	〇
19	COSM133190	p.E746_S752delinsI	c.2236_2256delinsATC				C	〇
19	COSM133191	p.E746_P753delinsIS	c.2236_2257delinsATCT				C	〇
19	COSM6966471	p.E746_A750delinsIP	c.2236_2248delinsATTC				C	〇
19	COSM133188	p.E746_S752delinsI	c.2236_2255delinsAT				C	〇
19	COSM51526	p.E746_T751delinsIP	c.2236_2253delinsATTCCT				C	〇
19	COSM13557	p.E746_A750delinsQP	c.2236_2248delinsCAAC				C	〇
19	COSM22999	p.E746_T751delinsQ	c.2236_2252delinsCA				C	〇
19	COSM6947327	p.E746_A750delinsP	c.2236_2250delinsCCT				C	〇
19	COSM13200	p.E746_P753delinsLS	c.2236_2257delinsCTCT				C	〇
19	COSM26513	p.E746_T751delinsS	c.2236_2251delinsT				C	〇
19	COSM4386694	p.I740_K745dup	c.2218_2235dup				C	〇
19	COSM144207	p.E746_A750delinsAP	c.2237_2248delinsCAC				C	〇
19	COSM6924852	p.E746_T751delinsAPS	c.2237_2251delinsCACCAT				C	〇
19	COSM6968288	p.E746_T751delinsVP	c.2237_2251delinsTCC				C	〇
19	COSM18426	p.E746_S752delinsV	c.2237_2256delinsTC				C	〇
19	COSM28623	p.E746_A750delinsVP	c.2237_2250delinsTCCCT				C	〇
19	COSM133193	p.E746_T751delinsV	c.2237_2253delinsTC				C	〇
19	COSM674057	p.E746_S752delinsV	c.2237_2256delinsTG				C	〇
19	COSM53205	p.E746_T751delinsVA	c.2237_2251delinsTGG				C	〇
19	COSM51524	p.E746_P753delinsVQ	c.2237_2258delinsTTCA				C	〇
19	COSM18421	p.E746_T751delinsVP	c.2237_2251delinsTTC				C	〇
19	COSM52935	p.E746_T751delinsVP	c.2237_2253delinsTTCCT				C	〇
19	COSM6980200	p.E746_T751delinsVS	c.2237_2251delinsTTT				C	〇
19	COSM26444	p.K745_E746insVPVAIK	c.2219_2236dup				C	〇
19	COSM255211	p.L747_P753del	c.2238_2258del				C	〇
19	COSM6978341	p.L747Qfs*16	c.2238_2247del				C	〇
19	COSM6974307	p.E746_A750delinsD	c.2238_2250delinsC				C	〇
19	COSM12421	p.L747_S752delinsQH	c.2238_2255delinsGCAACA				C	〇
19	COSM22944	p.L747_T751delinsP	c.2238_2251delinsGC				C	〇
19	COSM18428	p.E746_A750delinsDP	c.2238_2248delinsTC				C	〇
19	COSM9179903	p.L747_A750del	c.2239_2250del				C	〇
19	COSM24970	p.L747_K754del	c.2239_2262del				C	〇
19	COSM12420	p.L747_T751delinsQ	c.2239_2252delinsCA				C	〇
19	COSM51503	p.L747_T751delinsN	c.2239_2253delinsAAT				C	〇
19	COSM51501	p.L747_P753delinsQ	c.2239_2259delinsCAA				C	〇
19	COSM133196	p.L747_S752delinsQH	c.2239_2255delinsCAACA				C	〇
19	COSM4170220	p.L747_A750delinsP	c.2239_2250delinsCCG				C	〇
19	COSM6952818	p.L747Rfs*13	c.2239_2247delinsC				C	〇
19	COSM85891	p.L747_A755delinsAN	c.2239_2264delinsGCCAA				C	〇
19	COSM1667023	p.L747_K754delinsANKG	c.2239_2261delinsGCCAACAAGGG				C	〇
19	COSM23572	p.L747_T751delinsA	c.2239_2253delinsGCT				C	〇
19	COSM4170221	p.L747_A750delinsS	c.2240_2248del				C	〇
19	COSM7410537	p.L747*	c.2240delinsAAGAGAAGCAAC				C	〇
19	COSM1667026	p.L747_A755delinsSKG	c.2240_2264delinsCGAAAGG				C	〇
19	COSM20883	p.L747_K754delinsST	c.2240_2261delinsCGAC				C	〇
19	COSM6933365	p.L747_A755delinsSMS	c.2240_2263delinsCGATGT				C	〇
19	COSM18442	p.L747_R748delinsFP	c.2241_2244delinsCCCG				C	〇
19	COSM26439	p.A750_E758del	c.2248_2274del				C	〇
19	COSM5023004	p.A750_I759delinsPT	c.2248_2276delinsCCAAC				C	〇
19	COSM26440	p.A750_E758delinsP	c.2248_2273delinsCC				C	〇
19	COSM5023005	p.A750_I759delinsGS	c.2249_2277delinsGAAGT				C	〇
19	COSM26718	p.T751_A755del	c.2250_2264del				C	〇
19	COSM1667024	p.T751_I759delinsN	c.2250_2276delinsCAA				C	〇
19	COSM133200	p.T751_I759delinsS	c.2251_2276delinsTC				C	〇
19	COSM22945	p.T751_I759delinsS	c.2251_2277delinsTCT				C	〇
19	COSM23634	p.T751_E758del	c.2252_2275del				C	〇
19	COSM96856	p.T751_I759delinsN	c.2252_2276delinsA				C	〇
19	COSM24270	p.T751_I759delinsN	c.2252_2277delinsAT				C	〇
19	COSM22956	p.T751_I759delinsREA	c.2252_2277delinsGAGAAGCG				C	〇
19	COSM1667027	p.T751_I759delinsS	c.2252_2276delinsG				C	〇
19	COSM12410	p.T751Sfs*4	c.2252_2275delinsG				C	〇
19	COSM6978342	p.S752Efs*9	c.2253_2257del				C	〇
19	COSM6256	p.S752_I759del	c.2254_2277del				C	〇
19	COSM24269	p.P753_I759del	c.2258_2278del				C	〇
19	COSM12404	p.E746Nfs*15	c.2229_2252delinsAATTAAGA				C	〇
19	COSM24972	p.N756dup	c.2268_2270dup				C	〇

*Note:* Detectable EGFR gene mutations for each test in Exon 18 and 19. c: Variants detected as CDx, ref: Variants detected as reference information, 〇: Variants presumed detectable by design.

In conclusion, although ODxTT is an NGS test capable of analyzing many genes simultaneously, CGP should be performed early in patients with a high clinical suspicion of mutations, even after ODxTT, due to the risk of missing *EGFR* mutations that are not reportable variants.

## Author Contributions

T.T and N.S contributed to the conception of this study. N.S and Y.N contributed to data acquisition. N.S, T.T performed contributed to the interpretation of the results. N.S and T.T contributed to drafting the manuscript. N.S and T.T contributed to the manuscript revision. H.H and N.K supervised the study. All the authors have read and approved the final version of the manuscript.

## Conflicts of Interest

NS has received honoraria for lectures at his institution from AstraZeneca K.K., Chugai Pharmaceutical Co. Ltd., Novartis Pharma K.K., and Life Technologies Japan Ltd. TT reports fees paid to his institution for grants from Pfizer Japan Inc. and Takeda Pharmaceuticals, and personal fees for lectures from AstraZeneca K.K., Chugai Pharmaceutical Co. Ltd., Roche Diagnostics, Takeda Pharmaceuticals, MSD K.K., and Novartis Pharma K.K. KS received personal fees for lectures from Life Technologies Japan Ltd., Chugai Pharmaceutical Co. Ltd., Yodosha Co. Ltd., Qiagen Inc., Takeda Pharmaceutical Co. Ltd., and Nippon Kayaku Co. Ltd. KT reports personal fees for lectures from AstraZeneca K.K., Eisai Co. Ltd., Ono Pharmaceutical Co. Ltd., Chugai Pharmaceutical Co. Ltd., Taiho Pharmaceutical Co. Ltd., Kyowa Hakko Kirin Co. Ltd., Merck Biopharma Co. Ltd., Bristol‐Myers Squibb Co. Ltd., MSD K.K., Takeda Pharmaceutical Co. Ltd., and Novartis Pharma K.K. KK reports fees paid to his institution for grants from Chugai Pharmaceutical Co. Ltd. and Takeda Pharmaceutical Co. Ltd. and personal fees for lectures from Chugai Pharmaceutical Co. Ltd., AstraZeneca K.K., and Daiichi Sankyo Co. Ltd. SS reports fees paid to his institution for grants from Nippon Boehringer Ingelheim Co. Ltd. and personal fees for lectures from Amgen Inc. and AstraZeneca K.K. JT reports personal fees for lectures from Boehringer Ingelheim Japan Inc., Bristol‐Myers Squibb Co. Ltd., Chugai Pharmaceutical Co. Ltd., Daiichi Sankyo Co. Ltd., Eli Lilly Japan K.K., Janssen Pharmaceutical K.K., MSD K.K., Nihon Medi‐Physics Co. Ltd., Nippon Kayaku Co. Ltd., Taiho Pharmaceutical Co. Ltd., Ono Pharmaceutical Co. Ltd., Pfizer Japan Inc., AstraZeneca K.K., and Takeda Pharmaceuticals, and personal fees for participation on a Data Safety Monitoring Board or Advisory Board from AstraZeneca K.K. and AbbVie GK. KY reports fees paid to his institution for grants from Daiichi Sankyo Co. Ltd. and Boehringer Ingelheim Japan Inc., fees paid to his institution for royalties or licenses from Daiichi Sankyo Co. Ltd., personal fees for consultancy from Boehringer Ingelheim Japan Inc., personal fees for lectures from Amgen Inc., Chugai Pharmaceutical Co. Ltd., AstraZeneca K.K., Eli Lilly Japan K.K., Takeda Pharmaceutical Company Limited, and Merck Sharp & Dohme K.K., and patents pending with US20200115467 and US20200061031. KF reports fees paid to his institution for grants from Pfizer Japan Inc. and personal fees for lectures from Chugai Pharmaceutical Co. Ltd., KYORIN Pharmaceutical Co. Ltd., and IQVIA Inc. TM reports fees paid to his institution for grants from Boehringer Ingelheim Japan Inc. and Bridge Bio, personal fees for lectures from AstraZeneca K.K., Boehringer Ingelheim Japan Inc., Bristol‐Myers Squibb Company, Chugai Pharmaceutical Co. Ltd., Eli Lilly Japan K.K., Merck Sharp & Dohme K.K., Pfizer Japan Inc., Takeda Pharmaceuticals, Amgen Inc., Novartis Pharma K.K., Daiichi Sankyo Co. Ltd., and Thermo Fisher Scientific, and personal fees for participation on a data committee from Taiho Pharmaceutical Co. KN reports fees paid to his institution for grants from Nichirei Biosciences Corporation, NHO Osaka Minami Medical Center, West Japan Oncology Group, Nippon Boehringer Ingelheim Co., Hitachi Ltd., Otsuka Pharmaceutical Co., Thoracic Oncology Research Group, University Public Corporation Osaka, and Eli Lilly Japan K.K.; personal fees for consultancy from SymBio Pharmaceuticals K.K., Eli Lilly Japan K.K., and Otsuka Pharmaceutical Co. Ltd.; personal fees for lectures from Boehringer Ingelheim Japan, Yakult Honsha Co. Ltd., AstraZeneca K.K., Takeda Pharmaceutical Co. Ltd., Chugai Pharmaceutical Co. Ltd., Fujirebio Inc., Novartis Pharma K.K., Janssen Pharmaceutical K.K., Bristol‐Myers Squibb Co. Ltd., MSD K.K., Ono Pharmaceutical Co. Ltd., Daiichi Sankyo Inc., Pfizer Japan Inc., Invitae Japan K.K., Guardant Health, Nichirei Biosciences Inc., Amgen K.K., Maruho Co. Ltd., Merck Biopharma Co. Ltd., and Eli Lilly Japan K.K. HH reports fees paid to his institution for grants from IQVIA Services Japan K.K., Eisai Co. Ltd., Syneos Health Clinical K.K., EP‐CRSU Co. Ltd., EPS Corporation, Shionogi & Co. Ltd., Nippon Kayaku Co. Ltd., Otsuka Pharmaceutical Co. Ltd., Takeda Pharmaceutical Co. Ltd., GlaxoSmithKline K.K., MSD K.K., Sanofi K.K., Amgen Inc., Chugai Pharmaceutical Co. Ltd., Taiho Pharmaceutical Co. Ltd., Nippon Boehringer Ingelheim Co. Ltd., Bristol‐Myers Squibb Company, SRL Medisearch Inc., Janssen Pharmaceutical K.K., PRA Health Sciences Inc., CMIC Co. Ltd., Astellas Pharma Inc., Pfizer R&D Japan G.K., Ascent Development Services, Labcorp Development Japan K.K., Eisai Inc., Kobayashi Pharmaceutical Co. Ltd., Bayer Yakuhin Ltd., Pfizer Japan Inc., AstraZeneca K.K., AbbVie Inc., Daiichi Sankyo Co. Ltd., A2 Healthcare Corp., Novartis Pharma K.K., Eli Lilly Japan K.K., Merck Biopharma Co. Ltd., Medpace Japan K.K., Kyowa Kirin Co. Ltd., Japanese Gastric Cancer Association, Thoracic Oncology Research Group, Clinical Research Support Center Kyushu, West Japan Oncology Group, Japan Clinical Cancer Research Organization, Comprehensive Support Project for Oncological Research of Breast Cancer, EPS International Co. Ltd., Mebix Inc., Ono Pharmaceutical Co. Ltd., Mochida Pharmaceutical Co. Ltd., Covance Japan Inc., and Japan Clinical Research Operations Medical Research Support; personal fees for lectures from Ono Pharmaceutical Co. Ltd., Merck Biopharma Co. Ltd., Daiichi Sankyo Co. Ltd., 3H Clinical Trial Inc., AstraZeneca K.K., Novartis Pharma K.K., Chugai Pharmaceutical Co. Ltd., Bristol‐Myers Squibb Company, Eli Lilly Japan K.K., Amgen Inc., MSD K.K., Sysmex Corporation, Pfizer Japan Inc., Takeda Pharmaceutical Co. Ltd., Nippon Boehringer Ingelheim Co. Ltd., Janssen Pharmaceutical K.K., and Guardant Health Japan Corp.; personal fees for participation on a Data Safety Monitoring Board or Advisory Board from Bristol‐Myers Squibb Company, AbbVie Inc., Chugai Pharmaceutical Co. Ltd., Novocure K.K., AstraZeneca K.K., Daiichi Sankyo Co. Ltd., and Janssen Pharmaceutical K.K. during the conduct of the study. KN reports fees paid to his institution for grants from IQVIA Services Japan K.K., Syneos Health Clinical K.K., EPS Corporation, Nippon Kayaku Co. Ltd., EPS International Co. Ltd., Daiichi Sankyo Co. Ltd., Takeda Pharmaceutical Co. Ltd., MSD K.K., Ono Pharmaceutical Co. Ltd., Amgen Inc., Taiho Pharmaceutical Co. Ltd., EP‐CRSU Co. Ltd., Mebix Inc., Bristol‐Myers Squibb K.K., Janssen Pharmaceutical K.K., Pfizer R&D Japan G.K., Kobayashi Pharmaceutical Co. Ltd., Pfizer Japan Inc., Astellas Pharma Inc., Eisai Co. Ltd., AstraZeneca K.K., Mochida Pharmaceutical Co. Ltd., Labcorp Development Japan K.K. (Covance Japan Inc.), Japan Clinical Research Operations, Otsuka Pharmaceutical Co. Ltd., GlaxoSmithKline K.K., Sanofi K.K., Chugai Pharmaceutical Co. Ltd., Nippon Boehringer Ingelheim Co. Ltd., SRL Inc., Medical Research Support, Eli Lilly Japan K.K., Novartis Pharma K.K., CMIC Co. Ltd., Bayer Yakuhin Ltd., Shionogi & Co. Ltd., PRA Health Sciences Inc., and Ascent Development Services; personal fees for consultancy from Eli Lilly Japan K.K. and Ono Pharmaceutical Co. Ltd.; personal fees for lectures from Ono Pharmaceutical Co. Ltd., Amgen Inc., Nippon Kayaku Co. Ltd., AstraZeneca K.K., Chugai Pharmaceutical Co. Ltd., Eli Lilly Japan K.K., MSD K.K., Pfizer Japan Inc., Nippon Boehringer Ingelheim Co. Ltd., Taiho Pharmaceutical Co. Ltd., Bayer Yakuhin Ltd., Daiichi Sankyo Co. Ltd., Incyte Biosciences Japan, M3 Inc., Global Health Consulting Japan Co. Ltd., The Yomiuri Shimbun, Merck Biopharma Co. Ltd., TAIYO Pharma Co. Ltd., Takeda Pharmaceutical Co. Ltd., Life Technologies Japan Ltd., Neo Communication, Novartis Pharma K.K., Medical Mobile Communications Co. Ltd., Yodosha Co. Ltd., CMIC ShiftZero K.K., Japan Clinical Research Operations, CMIC Co. Ltd., Bristol‐Myers Squibb Company, Janssen Pharmaceutical K.K., Otsuka Pharmaceutical Factory Inc., and Hisamitsu Pharmaceutical Co. Ltd.; and patents planned, issued, or pending with Daiichi Sankyo Co. Ltd. The other authors declare no conflicts of interest.

## Supporting information


**FIGURE S1.** Patient Flow chart of 418 patients who underwent ODxTT.

## Data Availability

The data that support the findings of this study are available from the corresponding author upon reasonable request.
