# Supplementary material for: Detection of Overlooked Rare EGFR Mutations in Non‐small Cell Lung Cancer Using Multigene Testing
Source: Thorac Cancer. 2025 Feb 13;16(3):e70007. doi: 10.1111/1759-7714.70007 (PMC11825211; doi:10.1111/1759-7714.70007)
Supplement: Supplementary file 1 — FIGURE S1. Patient Flow chart of 418 patients who underwent ODxTT. [file TCA-16-e70007-s001.pptx]

## Slide 1
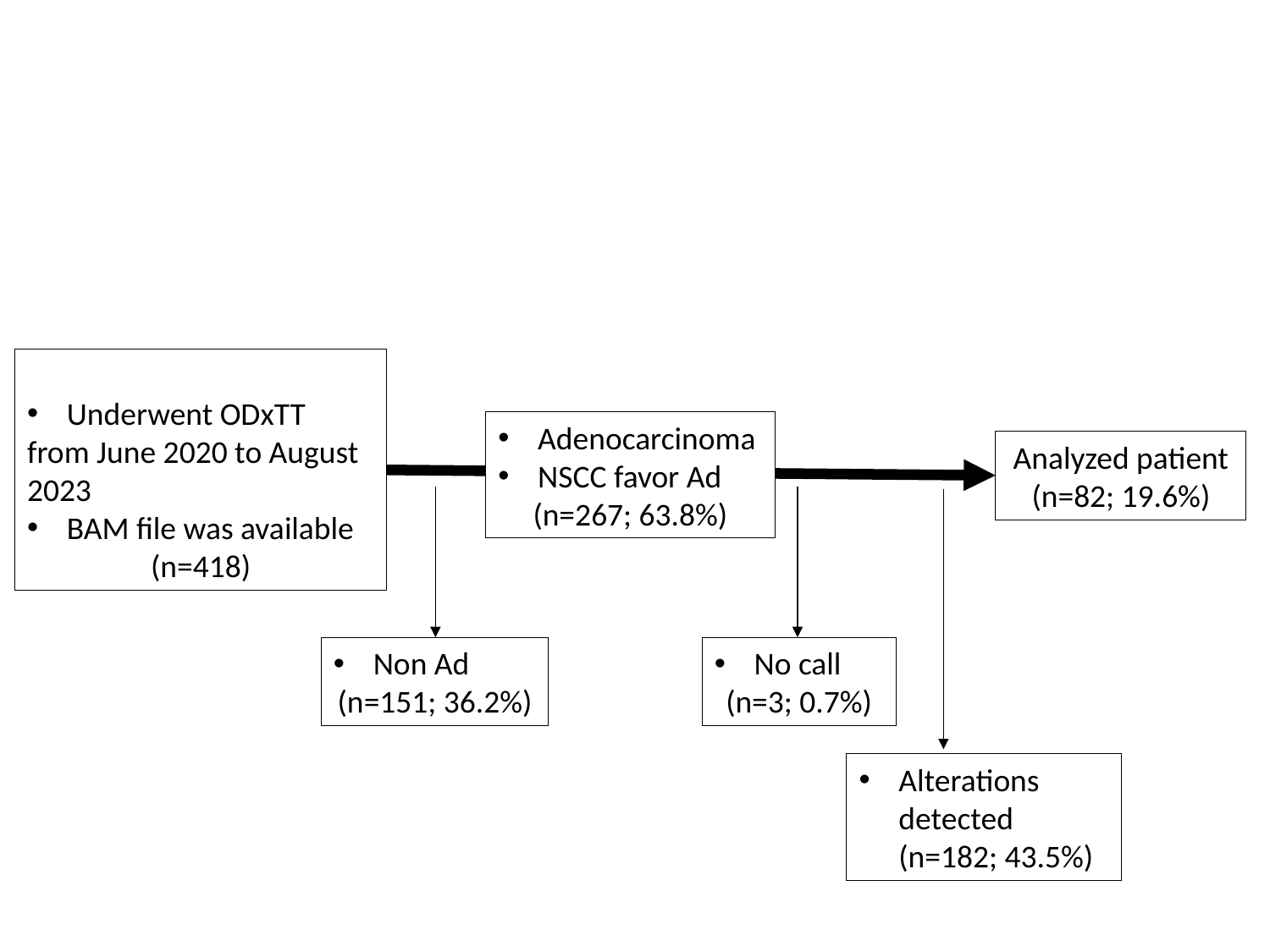

Underwent ODxTT
from June 2020 to August 2023
BAM file was available
(n=418)
Adenocarcinoma
NSCC favor Ad
(n=267; 63.8%)
Analyzed patient
(n=82; 19.6%)
Non Ad
(n=151; 36.2%)
No call
(n=3; 0.7%)
Alterations detected
(n=182; 43.5%)
